# BIC: a database for the transcriptional landscape of bacteria in cancer

**DOI:** 10.1093/nar/gkac891

**Published:** 2022-10-20

**Authors:** Kai-Pu Chen, Chia-Lang Hsu, Yen-Jen Oyang, Hsuan-Cheng Huang, Hsueh-Fen Juan

**Affiliations:** Graduate Institute of Biomedical Electronics and Bioinformatics, National Taiwan University, Taipei 106, Taiwan; Department of Medical Research, National Taiwan University Hospital, Taipei 100, Taiwan; Graduate Institute of Biomedical Electronics and Bioinformatics, National Taiwan University, Taipei 106, Taiwan; Institute of Biomedical Informatics, National Yang Ming Chiao Tung University, Taipei 112, Taiwan; Graduate Institute of Biomedical Electronics and Bioinformatics, National Taiwan University, Taipei 106, Taiwan; Department of Life Science, National Taiwan University, Taipei 106, Taiwan; Center for Computational and Systems Biology, National Taiwan University, Taipei 106, Taiwan

## Abstract

Microbial communities are massively resident in the human body, yet dysbiosis has been reported to correlate with many diseases, including various cancers. Most studies focus on the gut microbiome, while the bacteria that participate in tumor microenvironments on site remain unclear. Previous studies have acquired the bacteria expression profiles from RNA-seq, whole genome sequencing, and whole exon sequencing in The Cancer Genome Atlas (TCGA). However, small-RNA sequencing data were rarely used. Using TCGA miRNA sequencing data, we evaluated bacterial abundance in 32 types of cancer. To uncover the bacteria involved in cancer, we applied an analytical process to align unmapped human reads to bacterial references and developed the BIC database for the transcriptional landscape of bacteria in cancer. BIC provides cancer-associated bacterial information, including the relative abundance of bacteria, bacterial diversity, associations with clinical relevance, the co-expression network of bacteria and human genes, and their associated biological functions. These results can complement previously published databases. Users can easily download the result plots and tables, or download the bacterial abundance matrix for further analyses. In summary, BIC can provide information on cancer microenvironments related to microbial communities. BIC is available at: http://bic.jhlab.tw/.

## INTRODUCTION

The human microbiota massively lives, varies in our bodies, and is diverse in different body sides (1,2). It was estimated that a human body harbors more than three trillion bacterial members, similar to the number of human cells (3). Host–microbiome interactions impact multiple physiological processes and disease susceptibilities. The human microbiota plays an important role in human health, such as maintaining homeostasis, immunity and inflammation (4,5). Most microbial studies focus on the gut microbiome and related diseases, such as inflammatory bowel disease (IBD) and depression and anxiety (6). Furthermore, studies have shown that the microbial compositions are different and associated with cancer (7,8).

While many studies focus on the gut microbiome derived from patients’ stool (9–11), the bacteria that participate in the on-site tumor microenvironments remain unclear. Dohlman *et al.* and Poore *et al.* have acquired the bacteria expression profiles from RNA-seq, whole genome sequencing (WGS), and whole exon sequencing (WXS) in The Cancer Genome Atlas (TCGA) (12,13). However, the small-RNA sequencing data are not used. We developed an analytical approach using the small-RNA sequencing data of colorectal cancer (CRC) tissue samples to study cancer-associated microbiome in CRC and observed similar results compared to other studies using 16S rDNA sequencing (14).

There are certain benefits in using miRNA-seq compared to WGS, WXS, and RNA-seq. First, small RNAs (sRNAs) have been found to play regulatory roles in both bacteria and bacterial infectious diseases (15,16). Compared to WGS and WXS, sRNAs were transcribed and functional in either bacteria or hosts. Only a small fraction of total RNA was polyadenylated and appeared transiently in bacteria (17,18). In many RNA-seq studies, RNAs were extracted and reverse-transcribed to cDNAs through poly-A tails. Most bacterial RNAs without poly-A tails will be filtered in RNA-seq data. Compared to RNA-seq, miRNA-seq which is processed without poly-A filtering could have a chance to identify bacteria not found in RNA-seq.

**Figure 1.**
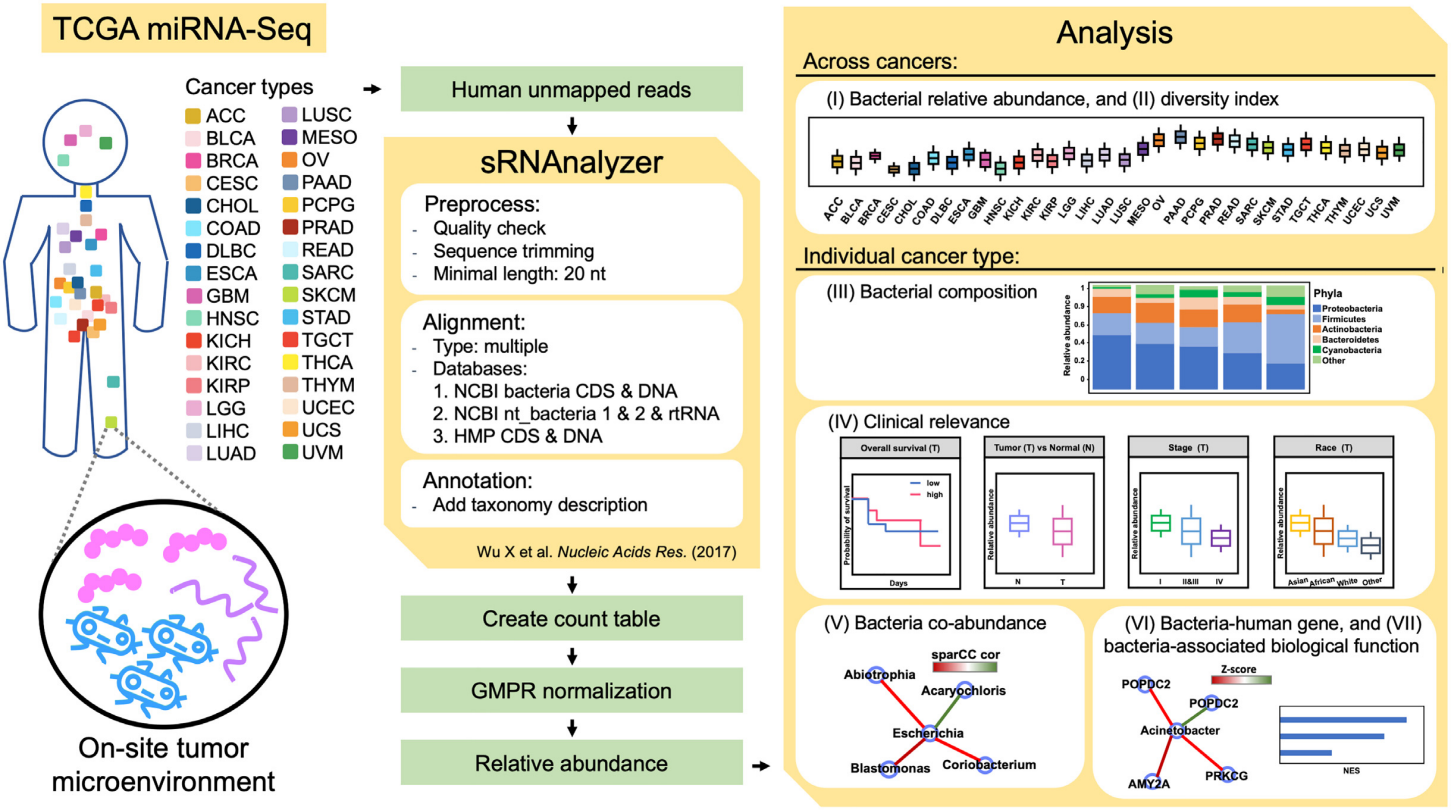
An overview of BIC analysis workflow. We downloaded miRNA-seq data from TCGA and used sRNAnalyzer for read processing. We parsed and merged count tables, conducted GMPR normalization, and produced the bacterial relative abundance matrixes of each taxonomic level in our scripts. We provide seven modules in the BIC Analyses panel for users to query and download results.

Using TCGA miRNA sequencing data, we evaluated tissue-resident bacterial abundance in 32 types of cancer. We aligned unmapped human reads to bacterial references by sRNAnalyzer and merged them for each taxonomic rank of 32 cancer types (14,19). The bacterial relative abundance and sample diversity were compared across different cancer types. We parsed all the data and developed the BIC database for the transcriptional landscape of bacteria in cancer. BIC provides the following information: (i) relative abundance of bacteria, (ii) bacterial diversity, (iii) bacterial composition, (iv) clinical relevance, (v) bacterial co-abundance network, (vi) bacteria-correlated human gene expression network and (vii) bacteria-associated biological function (Figure 1). Users can easily query and browse the analysis plots and result tables, or download the bacterial expression matrices for further analyses.

## DATA COLLECTION

The TCGA miRNA-seq BAM files were retrieved from the NCI Genomic Data Commons (GDC) using the GDC Data Transfer Tool (20). Human RNA expression profiles (EBPlusPlusAdjustPANCAN_IlluminaHiSeq_RNASeqV2.geneExp.tsv), tumor stages, races, survival events, and time (TCGA-CDR-Supplemental Table S1.xlsx) were downloaded from the Supplemental Data in PanCanAtlas Publications (https://gdc.cancer.gov/about-data/publications/pancanatlas). The gene symbols in the RNA expression profiles were renamed according to org.Hs.eg.db (version 3.6.0) (21). Only samples from primary tumors and their adjacent normal tissues were used. We acquired the biospecimen information using TCGAbiolinks (version 2.17.3) (22).

## DATA PROCESSING AND INTEGRATION

### Bacteria relative abundance matrixes

**Figure 2.**
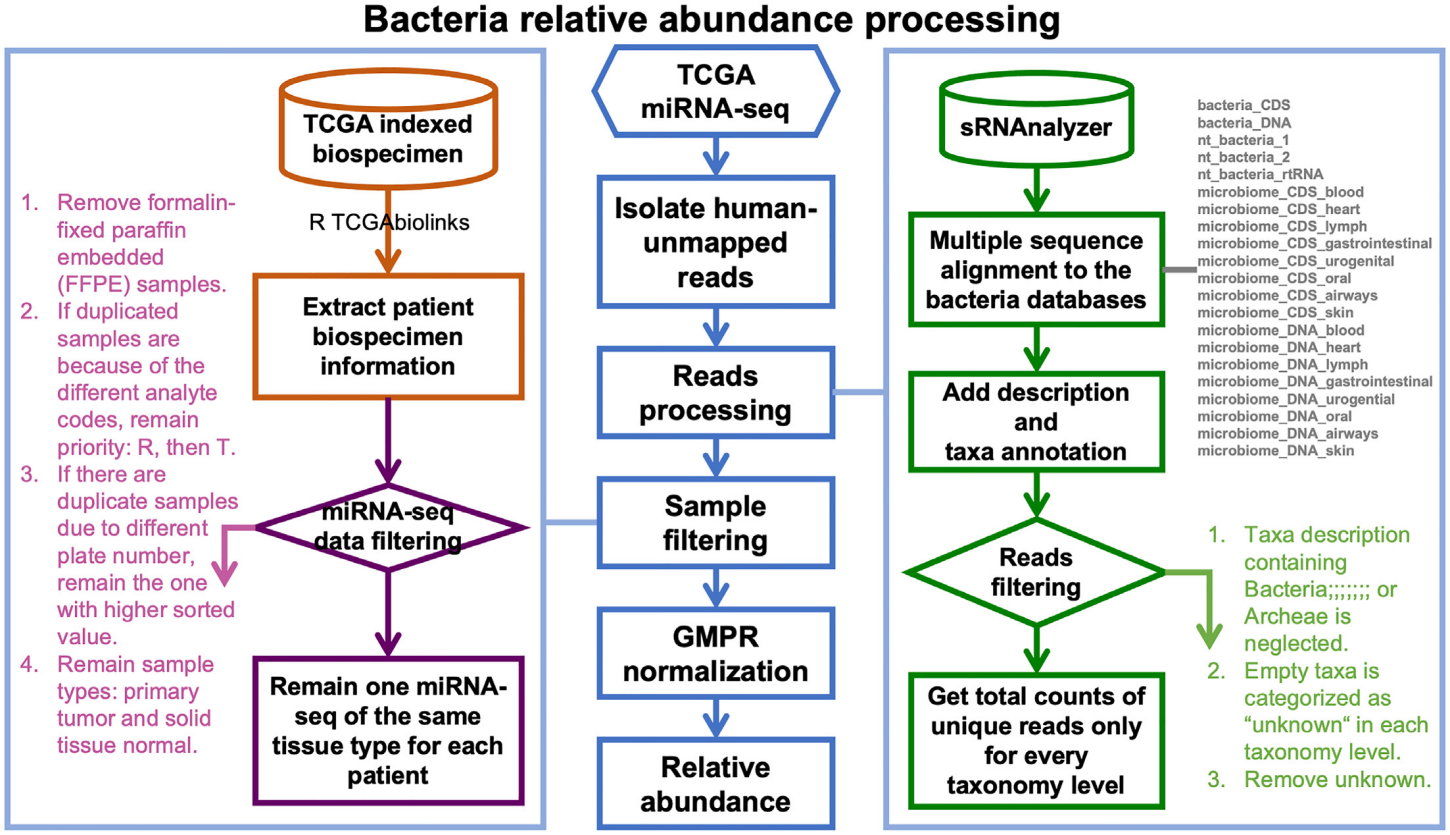
Workflow of data processes to produce bacterial relative abundance matrixes.

We used SAMtools (version 1.3.1) to extract the unmapped reads from human miRNA-seq BAM files and stored them in FASTQ format (23). sRNAnalyzer scripts (‘preprocess.pl’, ‘align.pl’, ‘desProfile.pl’ and ‘taxProfile.pl’) were used for read preprocessing, alignment, taxonomy annotation (19). We set the minimal read length to 20 nucleotides and mapped the reads to multiple references, but did not allow any mismatch to obtain the highest alignment accuracy. The references used in alignment were provided by sRNAnalyzer, including CDS and DNA of bacteria, nt_bacteria, and microbiomes. After taxonomy annotation by the sRNAnalyzer scripts, we reassigned the reads mapped to multiple species to their common higher-level taxa and generated the read matrixes at different taxonomic levels (14). The processed read counts in each data processing step are summarized in Supplementary Figure S1. We identified 1617 genera, 303 families, 126 orders, 56 classes, and 47 phyla from 10362 samples (9709 patients) across 32 cancer types. Since the count matrixes were sparse, we applied the geometric mean of pairwise ratio (GMPR), a robust normalization method for zero-inflated data, to produce normalized count tables (24). To keep all 10 362 samples, the intersection numbers of the phylum, class, order, family, and genus count tables were set to 3, 5, 5, 6 and 5, respectively. Normalized count matrices were transformed into relative abundance matrices. The relative bacterial abundance of each taxonomy level was used for all subsequent analyses in BIC. An overview of these processes is shown in Figure 2. Detailed bacterial references and processing scripts are available on GitHub.

### Precomputed analysis data and database construction

**Figure 3.**
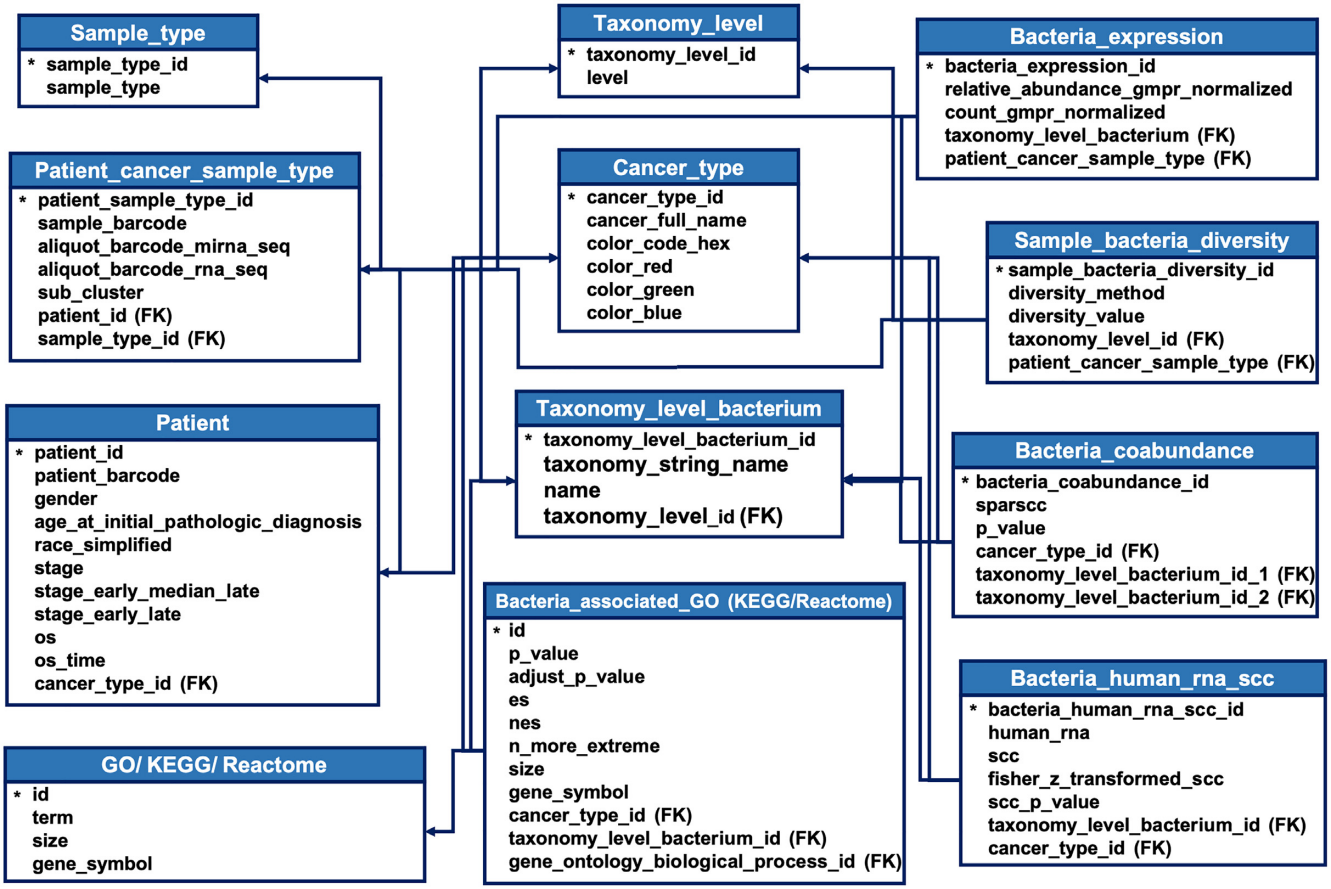
Data tables saved in PostgreSQL. All the precomputed analysis results were saved in PostgreSQL and the primary key of each table was labeled with a star symbol (*).

Based on bacterial relative abundances, we calculated the bacterial diversity in each taxonomy level for every kind of cancer. Vegan (version 2.5–7, https://CRAN.R-project.org/package=vegan) was used to calculate the Shannon, Gini-Simpson, and inverse Simpson indices (25). Bacteria with a prevalence (nonzero count) of ≥20% in the individual type of cancer were used to analyze the co-abundance of bacteria, the correlation with host gene expression, and the associated biological function. We applied SparCC, a method designed for compositional data, to calculate bacterial co-abundance relationships and establish the co-abundance networks for individual cancer types (26,27). The function sparccboot in SpiecEasi (version 1.1.0) was used to acquire SparCC correlation coefficients and empirical p-values of the bacterial co-abundance with 10 000 times of bootstraps (28). Spearman correlation coefficients (SCC) were calculated for the bacterial correlation with human gene expression using common samples between bacteria and tissue transcriptome data. Only human genes that were measured with nonzero counts in ≥20% of the samples were considered. To correct for the sample size effect, we applied Fisher's z-transformation for SCC. To reveal the possible biological processes in which the queried bacteria are involved, we performed gene set enrichment analysis (FGSEA, version 1.12.0) for bacteria-correlated gene expression ranked in the descending order of the corrected *z*-score. The gene sets of biological processes annotated by Gene Ontology (GO, c5.go.bp.v7.2) (29), KEGG (c2.cp.kegg.v7.5.1) (30) and Reactome (c2.cp.reactome.v7.5.1) (31) were downloaded from the Molecular Signatures Database (32,33). These analyses were performed with R scripts (version 3.6.0) (34) and all the precomputed analysis data are stored in PostgreSQL (version 13.3). The tables deposited in PostgreSQL are shown in Figure 3.

### Web application framework

The BIC web application framework (Supplementary Figure S2) was constructed using Python (version 3.6.8) (35) and Django (version 3.2.3, https://djangoproject.com). The analyses of clinical relevance were performed under Django, including overall survival and bacterial abundance comparison of different groups, such as tumor (T) versus adjacent normal (N), tumor stages and races. The survival analysis was implemented using lifelines (version 0.26.3) (36). Calculations of statistical *P*-values (Kruskal–Wallis and Wilcox ranksum tests) in different groups were implemented by kruskal and ranksums in scipy (version 1.5.4) (37). Plots were produced by bokeh (version 2.3.3, http://www.bokeh.pydata.org).

## USER INTERFACE AND USE CASES

**Figure 4.**
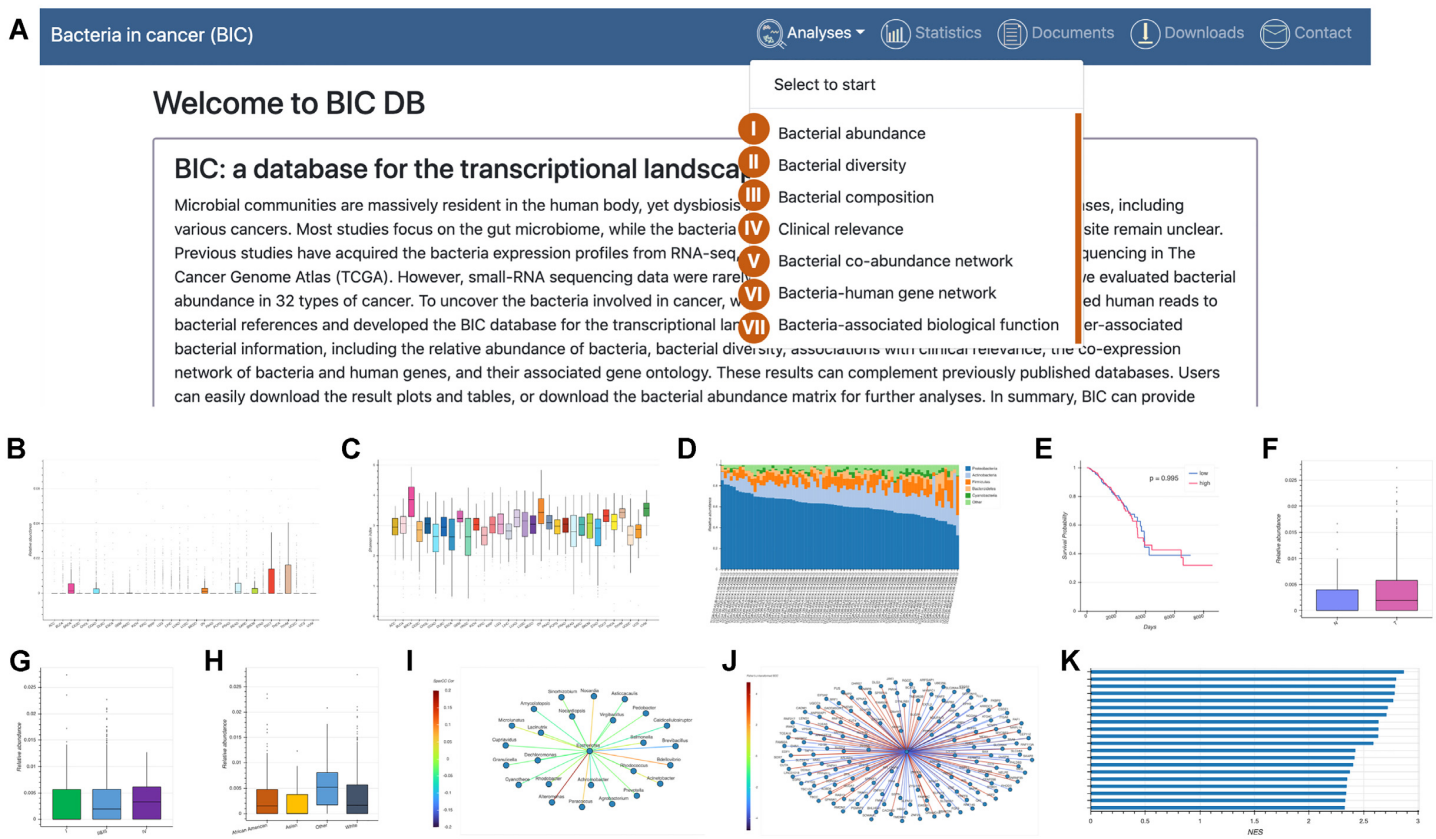
BIC user interface and analysis modules. (**A**) The user interface and analysis modules. (**B**, **C**) Modules I and II show the relative abundance and diversity of bacteria across cancers. (**D**) Module III shows the bacteria composition. (**E**–**H**) Module IV shows the clinical relevance, such as overall survival, and relative abundance compared in different groups (tumor versus adjacency normal; tumor stages; races). (**I**) Module V shows the bacteria co-abundance network. (**J**) Module VI shows the bacteria-correlated human gene expression network. (**K**) Module VII shows the bacteria-associated biological functions.

Figure 4 shows the user interface and all the analyses provided by BIC. Modules I and II enable users to query the bacterial relative abundance and diversity indexes or evenness of the selected taxonomy level across all cancer types. Modules III to VII allow users to find the bacterial composition, clinical relevance, co-abundance, correlated human gene expression, and inferred biological processes of the queried bacteria under specified taxonomy level of the selected cancer type. Users can easily save the output plots and tables for their queried analyses.

**Figure 5.**
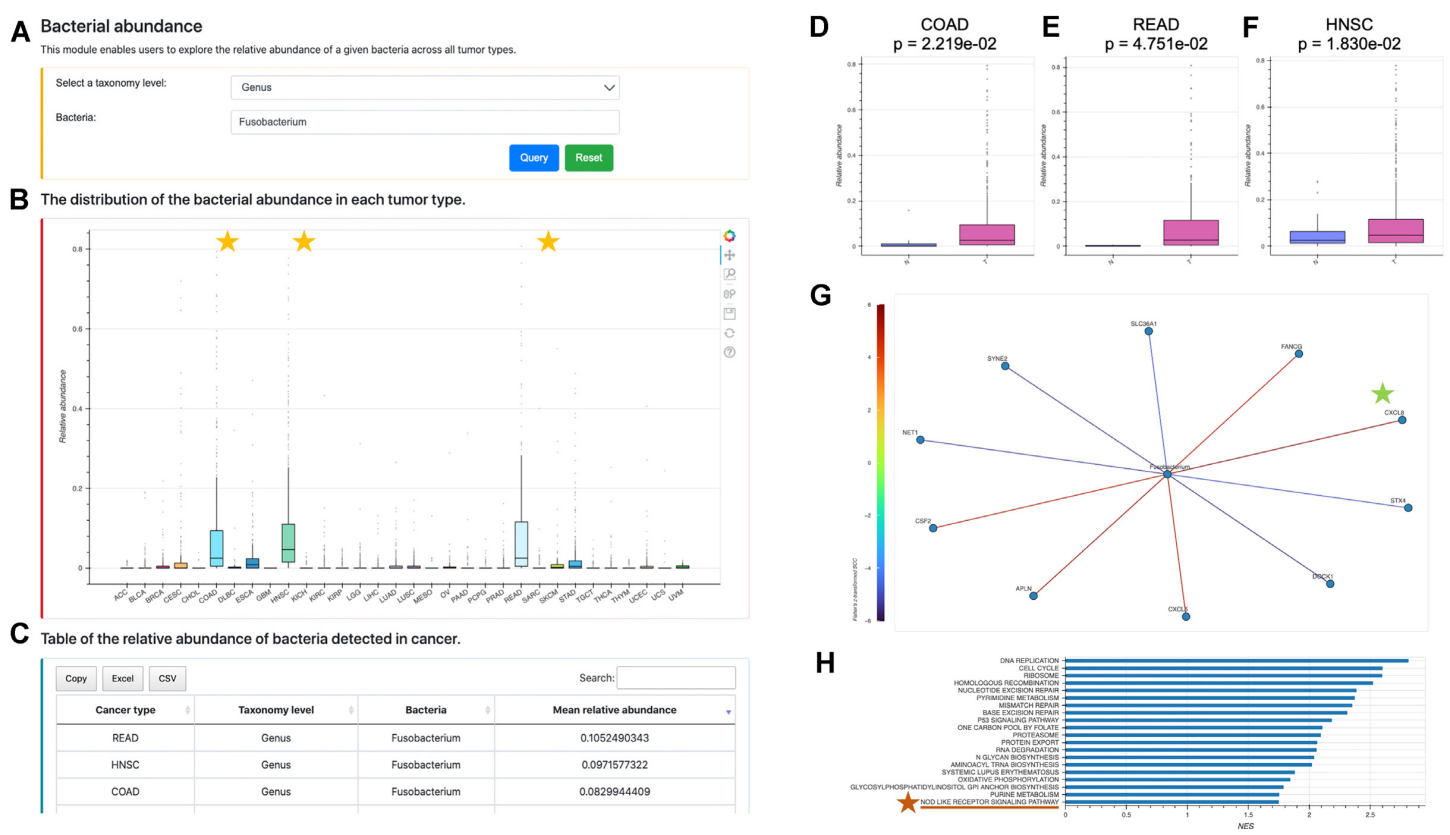
Query examples of the analysis modules in BIC. (**A**–**C**) Query of *Fusobacterium* at the genus level and the output distribution plot and tables for the abundance of *Fusobacterium* across cancer types in the bacteria abundance module. (**D**–**F**) The relative abundance of *Fusobacterium* in tumor is significantly higher than in the adjacent normal tissues in COAD, READ and HNSC with the clinical relevance module. (**G**) The bacteria-human gene network module displays the *z*-scores between *Fusobacterium* and the top 10 correlated genes in COAD. (**H**) The bacteria-associated biological function module displays the top 20 *Fusobacterium*-associated KEGG pathways in COAD.

Figure 5 illustrates an example of how users can investigate the genus *Fusobacterium* in cancer. For CRC and head and neck cancer, *Fusobacterium* is known to be associated with cancer progression (38,39). With the Bacterial abundance module, users can query *Fusobacterium* at the genus level (Figure 5A) and observe that the relative abundances of *Fusobacterium* are remarkably high in COAD (colon adenocarcinoma), READ (rectum adenocarcinoma), and HNSC (head and neck squamous cell carcinoma) (Figure 5B, C). Furthermore, the Clinical relevance module shows that *Fusobacterium* is more abundant in tumor than in adjacent normal tissues in these three types of cancer (Figure 5D–F). In the Bacteria-human gene network module, *CXCL8* is the top gene positively correlated with *Fusobacterium* in COAD (Figure 5G). *CXCL8* has been found to play an important role in CRC (40,41). With the Bacteria-associated biological function module, users can view the most significant KEGG pathways correlated with the abundance of *Fusobacterium* in COAD (Figure 5H). Among many cancer-related pathways, the NOD-like receptor signaling pathway has previously been reported to be related to the onset of CRC (40,41).

## CONCLUSION

We have developed a user-friendly database, BIC, for bacterial profiles derived from TCGA miRNA-seq data in 32 types of cancer. BIC allows comparisons of the relative abundance and diversities of bacteria in different types of cancer. BIC also provides the bacterial composition, clinical relevance, co-abundance network, correlated human gene expression network, and associated gene ontologies, for each type of cancer. With the comprehensive characterization of bacteria in tissues of different cancers, BIC can greatly facilitate the exploration of bacterial functions and mechanisms in tumor microenvironments. We believe that our database will be a valuable resource for understanding the interactions between humans and microbes in cancer formation.

## DATA AVAILABILITY

BIC is freely accessible at: http://bic.jhlab.tw/. The entire BIC data collection can be downloaded from the website. The source codes of BIC data processing, database construction, and web application are available at GitHub https://github.com/Kai-Pu/BIC_production.

## Supplementary Material

gkac891_Supplemental_File
