# Supplementary material for: BIC: a database for the transcriptional landscape of bacteria in cancer
Source: Nucleic Acids Res. 2022 Oct 20;51(D1):D1205–11. doi: 10.1093/nar/gkac891 (PMC9825443; doi:10.1093/nar/gkac891)
Supplement: gkac891_Supplemental_File [file gkac891_supplemental_file.pdf]

SUPPLEMENTARY DATA

BIC: a database for the transcriptional landscape of bacteria in cancer

Kai-Pu Chen, Chia-Lang Hsu, Yen-Jen Oyang, Hsuan-Cheng Huang, Hsueh-Fen Juan

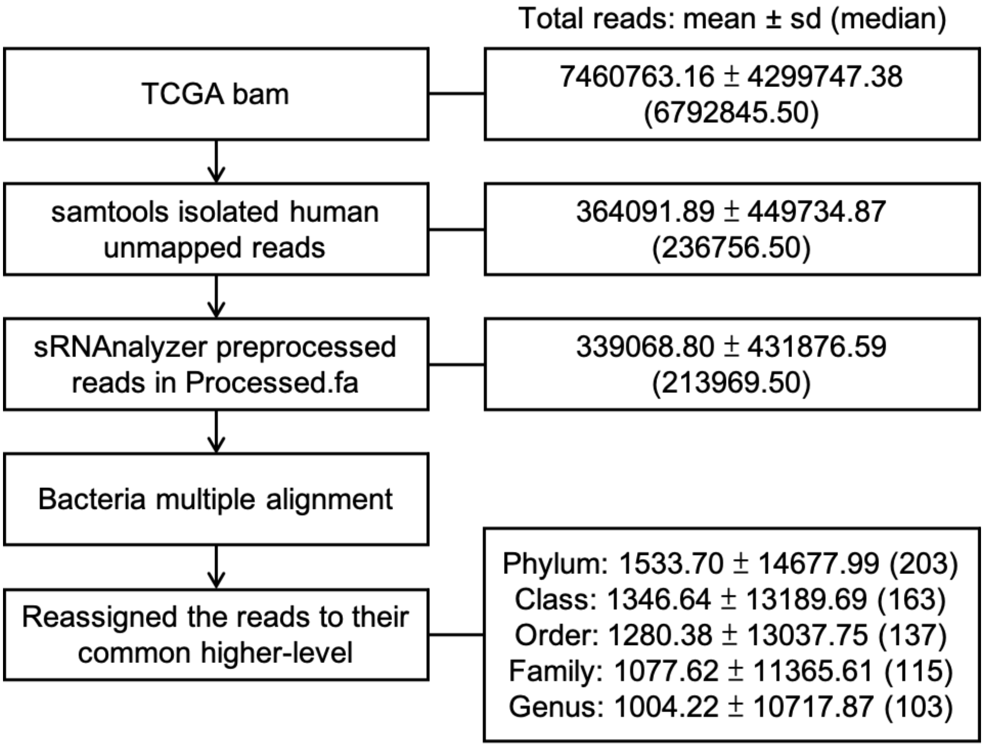

Supplementary Figure S1. Workflow and summary statistics of the bacterial read counts along the data processing pipeline. The average, standard deviation and median of processed total reads in each step are presented.

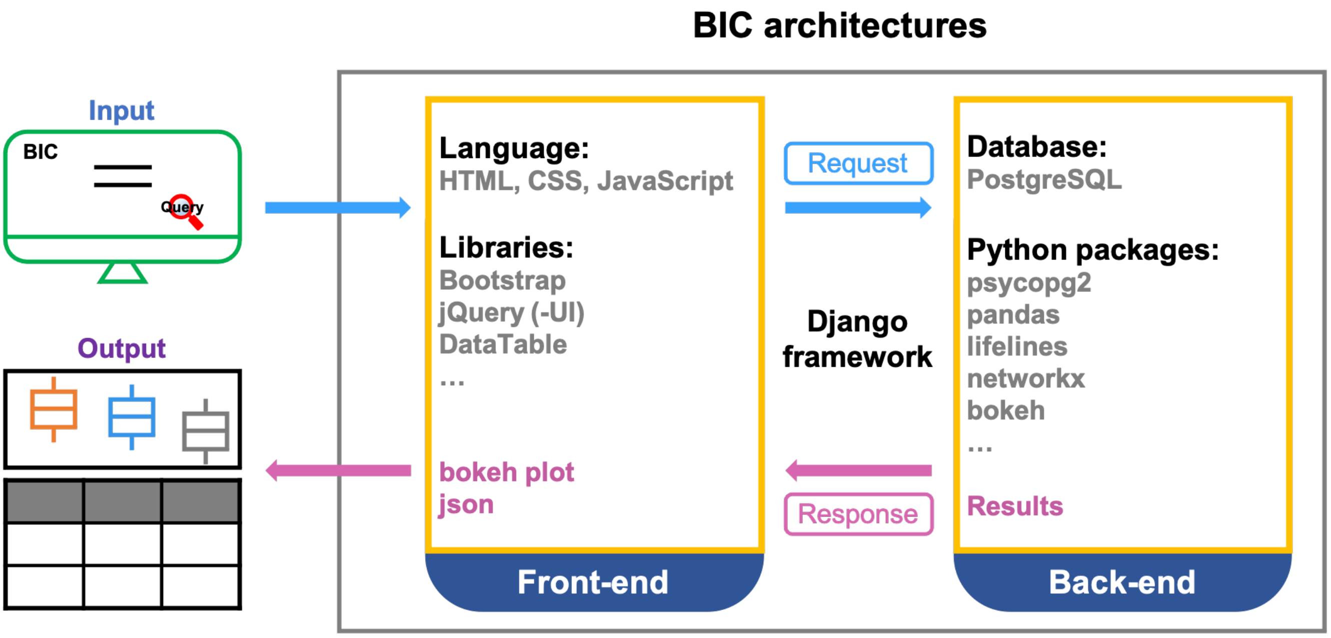

Supplementary Figure S2. BIC database framework. BIC is implemented in the Django framework. We created web content in HTML, styled in CSS, animated with JavaScript, and some plugins. We saved the precomputed data in PostgreSQL and retrieved and used it in Django views. We used Python packages to compute statistics and produce outputs of plots and tables.
